# Development of a Soft Sensor for Flow Estimation in Water Supply Systems Using Artificial Neural Networks

**DOI:** 10.3390/s22083084

**Published:** 2022-04-18

**Authors:** Robson Pacífico Guimarães Lima, Juan Moises Mauricio Villanueva, Heber Pimentel Gomes, Thommas Kevin Sales Flores

**Affiliations:** 1 Technology Center (CT), Postgraduate Program in Mechanical Engineering (PPGEM), Campus I, Federal University of Paraiba (UFPB), Joao Pessoa 58058-600, PB, Brazil; 2 Automation Coordination (CAUT), Federal Institute of Pernambuco (IFPE), Ipojuca 55590-000, PE, Brazil; 3 Renewable and Alternatives Energies Center (CEAR), Electrical Engineering Department (DEE), Campus I, Federal University of Paraiba (UFPB), Joao Pessoa 58058-600, PB, Brazil; jmauricio@cear.ufpb.br (J.M.M.V.); thommas.flores@cear.ufpb.br (T.K.S.F.); 4 Technology Center (CT), Department of Civil and Environmental Engineering (DECV), Campus I, Federal University of Paraiba (UFPB), Joao Pessoa 58058-600, PB, Brazil; heberp@uol.com.br

**Keywords:** indirect measurement, soft sensor, water supply systems, artificial neural networks

## Abstract

A water supply system is considered an essential service to the population as it is about providing an essential good for life. This system typically consists of several sensors, transducers, pumps, etc., and some of these elements have high costs and/or complex installation. The indirect measurement of a quantity can be used to obtain a desired variable, dispensing with the use of a specific sensor in the plant. Among the contributions of this technique is the design of the pressure controller using the adaptive control, as well as the use of an artificial neural network for the construction of nonlinear models using inherent system parameters such as pressure, engine rotation frequency and control valve angle, with the purpose of estimating the flow. Among the various contributions of the research, we can highlight the suppression in the acquisition of physical flow meters, the elimination of physical installation and others. The validation was carried out through tests in an experimental bench located in the Laboratory of Energy and Hydraulic Efficiency in Sanitation of the Federal University of Paraiba. The results of the soft sensor were compared with those of an electromagnetic flux sensor, obtaining a maximum error of 10%.

## 1. Introduction

Water is a natural resource of fundamental importance for the survival of humans and other living beings, and a good as relevant as this must be preserved in relation to its conscious use. The problem of water waste, not only in domestic waste (excessive use of water to wash sidewalks, time-consuming cleanliness with open registers, etc.) but mainly in waste from the point of view of supply systems (leakage in pipes, for example) makes the obstacle much higher. The feasibility of means that mitigate waste through an efficient distribution to a given location is extremely important. Thus, in the context of water distribution to the population, there is a need for an efficient resource distribution system capable of meeting urban and rural demands. In this way, a water supply system is defined as the set of works, equipment and services intended to supply drinking water to a community for the purposes of domestic consumption, public services, industrial consumption and other utilities [1]. This water supplied by the system must be in sufficient quantity and of the best quality from a physical, chemical and bacteriological point of view.

A water supply system (WSS) represents the entire process of supplying treated water, ranging from obtaining it to its use by the population. For this, a WSS uses a set of equipment, works and services, whose objective is to supply the water demand of a given population. Thus, the typical elements of a WSS are catchment (from a spring), pumping station, pipeline, water treatment station (WTS), reservoirs, distribution network and finally household connections (consumers).

The water stored in the distribution reservoir after the intake, elevation/adduction and WTS steps can be pressurized in the pipeline in two ways: by gravity and/or by a motor-pump set. By gravity, water is transported to the consumer without the need to use electricity. On the other hand, in the second case, an artificial impulsion system is needed, that is, by a hydraulic motor-pump coupled to an electric motor and/or by means of a booster (BST) [1].

In many practical cases, the non-linearity of the topography of a region causes the water pumping systems to operate in such a way as to meet these unevennesses, as in cases where there are low areas (topographic regions with low topographic elevation) and high areas (topographic regions with high topographic elevation in relation to low areas). In the case of the high zone, the pumping station, located in the low zone, must increase the pumping head to supply the high zone region. However, this increase causes an excess of pressure in the low zone and, therefore, tends to increase leakage losses and maintenance costs due to pipe rupture [1,2].

To solve excess pressure in the low zone and avoid damage to pipes and other installations, there are some solutions to be adopted: one of them is the installation of control valves (pressure-reducing valves or PRVs), automatic or not, with the purpose of modifying the pressures at certain points in the network. This solution is usually adopted, as pumps operating in water supply systems generally work at their nominal speed.

Another alternative is the application of mechanisms to control the rotation speed of electric motors and consequently the hydraulic energy supplied by the pumps. For this purpose, frequency inverters are generally adopted [2,3]. These devices convert a voltage into another with desired (controlled) amplitude and frequency. When used to drive a motor-pump set, the frequency inverter can manage the electrical and hydraulic parameters of a pumping station, because by regulating the rotation speed, it also regulates the head (pressure), flow and associated electrical power [4]. The energy released to promote the increase in flow and hydraulic pressure depends on the rotation speed of the pump set.

Several techniques can be used to promote the control of the frequency inverter applied to WSS for pressure management to keep it stable and contribute to the reduction in losses (electrical and hydraulic). The benefits of pressure control are to increase the useful life of pipes and accessories, increase system reliability, reduce hydraulic transients and reduce wasted electrical energy [1]. For a supply system to have a satisfactory technical and economic performance, it is necessary to perform pressure control. The uniformity of pressure in the distribution network, through this control, reduces the frequency of ruptures, excessive water consumption induced by pressure and the volume lost in leaks.

Several technologies can be used to measure the main physical quantities in WSS. The flow rate, which is of greater interest in this work, can be measured using technologies such as electromagnetic and ultrasonic, among others [5,6]. Other techniques can also be used to measure the flow, such as the equations that characterize the operation of a pump (pressure–flow curve). However, the use of this estimation method requires constant system calibrations, since they are based on the constructive characteristics of the pump, which wears out during operation, varying its characteristics over time [7].

This work describes the application of the adaptive control technique in a water supply system that emulates two regions with different topographic elevations. The control technique mentioned above acts as an aid in obtaining the necessary parameters for the validation of a virtual instrument, called a soft sensor, used to estimate the flow in this water supply system in the artificial neural network (ANN). The main contributions of the proposed architecture are summarized as follows: (a) the indirect measurements carried out through the soft sensor do not require the use of real flow instruments, providing savings in the acquisition of these instruments and eliminating the complexity of their installation [5,6]; (b) the construction of an ANN-based indirect reconstruction block, which allows the construction of nonlinear models of the pressure inputs, opening and closing angle of control valves, and the frequency of the inverter with the flow output; and (c) the development of virtual sensors or soft sensors with dynamic response to input variables [7].

## 2. Related Work

An alternative method for estimating the flow can be derived from the equations that characterize the operation of a pump (pressure–flow curves). However, the use of this estimation method requires a calibration constant, as they are based on the constructive characteristics of the pump, which wears out during operation. In [7] a system with feedback using ANN and controller using fuzzy logic was proposed for indirect estimation of flow in a water supply network. The authors used a MISO (multiple inputs single output) system to estimate the flow rate based on the rotation frequency of a motor-pump set and the pressure existing in the plant. The results were satisfactory, reaching the objective proposed in the tests to which the technique was tried. However, the authors did not submit the final product to tests that contained entries with outliers or noise that could compromise the efficiency of the technique in the face of these adversities and did not insert other entries for analysis. The study was limited to a MISO-type system. In [8], the authors discussed the possible applicability of soft sensors in industry using the characteristics of process data that are critical for the development of data-based virtual instruments. These characteristics are common to many fields in the process industry, such as the chemical industry, bioprocess industry, steel industry, etc. The focus of the work, of a theoretical nature, was evident in data-based soft sensors because of their growing popularity and potential. In [9], the authors proposed a NARX (nonlinear auto-regressive with exogenous inputs) ANN model for real-time prediction and control in a WSS application. The model developed estimated time–variable consumption demand in an equivalent way, exploring operational data of pressure and flow in real time and historically, establishing a functional relationship between the main variables of the network based on pressure. In addition, a training scheme for ANN with a combination of offline data training and online data training was proposed. The results showed that the model was considered applicable and satisfactory in terms of tracking and predicting network failures. Although the performance was satisfactory and the research objectives were achieved according to the authors, they did not implement the technique with other variables associated with a typical WSS, such as rotation frequency of motors that drive water, pressure-reducing valves, solenoid valves, etc., and did not promote a comparative study between some ANNs to verify the efficiency among them for the purpose of the study. It is also not described in the work if the system was validated with disturbances in the inputs or spurious signals that could compromise the efficiency of the system in the face of these undesirable conditions.

In [10] a methodology was proposed to detect false data and replace missing or false data (data reconstruction according to the authors) in flow data measurements in a WSS located in the city of Barcelona, Spain. Using ANN MLP BP (Multilayer Multilayer Perceptron Backpropagation) and Genetic Algorithms, the authors modeled time series generated from measurements of flow transducers located in the plant and used confidence intervals (values within a pre-established acceptable range) to validate the information. The authors, however, did not carry out comparative studies between other ANNs to verify which one would be more efficient in the applicability of the research. Furthermore, it is not described in the publication whether the implemented system experienced disturbances in the inputs, outliers or spurious signals that could compromise the efficiency of that system. In [11] studies were carried out on the predictability of future water demands in a distribution network (located in the city of Laminga in Nigeria) using ANN. They used NeuNETPro 2.3 software and developed a model based on historical water demand records using 15 pressure demand nodes. The results obtained served for the model to perform the control and supervision of the hydraulic parameters of the demand nodes and reduced the cost of water production. Pressure estimators were used as alternatives to the complex mathematical models and nonlinearities found in the plant. However, the authors did not use other estimation parameters to verify if the developed product could achieve greater optimization and the work does not mention tests with spurious signals in certain inputs. In [12] a soft sensor was implemented for estimating the total amount of phosphorus and chemical oxygen demand (COD) in streams and tributaries of an effluent treatment plant in a small Norwegian municipality. A mathematical model was developed identifying statistical correlations to feed the virtual sensor data. The data obtained with the virtual measurements were compared with measurements of the variables, demonstrating satisfactory results in relation to the estimation of the measured quantities.

In [13], a robotic replica of a human spinal column was implemented and printed in 3D to include an artificial disc implant and equipped with a set of magnetic soft sensors. This replica aimed at a new approach to allow surgeons to visualize the post-operative effects of an artificial disc implant in a specific way for a given patient before performing surgery, minimizing post-operative effects. Benchtop experiments showed that the array of magnetic soft sensors was able to accurately detect the location and amplitude of forces exerted on the analyzed column, which were successfully classified by four different machine learning algorithms: support vector machine (SVM), k-nearest neighbor (KNN), random forest (RF) and artificial neural network (ANN). The RF and RNA algorithms were able to classify load locations applied at 3.25 mm distance with 98.39% ± 1.50% and 98.05% ± 1.56%, respectively. In addition, the ANN had an accuracy of 94.46% ± 2.84% to classify the place where a load of 10 g was applied. The replica of the spine implanted by artificial disk was subjected to flexion and extension by a robotic arm. Five different spine postures were successfully classified with 100% ± 0.0% accuracy with the ANN using the soft magnetic sensor array.

Contextualizing for studies using adaptive controllers, in [14], a simultaneous control of altitude and vibrations was developed for a spacecraft in a three-dimensional space, subject to disturbances from inputs and unknown faults. The system dynamics were modeled as an infinite dimensional space, using partial differential equations. The control strategy, of adaptive type, was implemented to suppress the vibrations of the spacecraft’s flexible panel during altitude stabilization. Several simulations were carried out and the results obtained proved the efficiency of the implemented adaptive control proposal. In [15], a robust adaptive controller for flexible riser vibratory systems was developed, in which these systems were affected by input nonlinearities and unknown external disturbances. An auxiliary system was built and tuned to develop robust adaptive threshold control to constrain vibrational displacement and eliminate the effect of input nonlinearities. In addition, an adaptive upper bound boundary perturbation law was developed along with a vibration control strategy to estimate the magnitude of the unknown boundary disturbances. Simulation results with the developed techniques were performed and the results obtained corroborated a satisfactory performance of the implemented adaptive control.

In this work, the conception of a virtual instrument is proposed, called a soft sensor, capable of measuring (indirectly) the flow from several input parameters commonly used in water supply systems. The experimental bench used consists of a water supply system that emulates a region with a topographical dimension. Thus, the aim is, with the implementation of the soft sensor, to evaluate the performance of the virtual instrument, through various system performance scenarios, such as no control action on the plant, with control action on the plant as well as the insertion of disturbances and noise in the system. Noise can occur in real instruments commonly found in water supply systems and can compromise the correct measurement of physical quantities. Thus, the aim is, as one of the contributions of this work, to fill in some gaps found in some works pertinent to the topic.

## 3. Theoretical Background

### 3.1. Soft Sensor

In recent decades, soft sensors have established themselves as a valuable alternative to traditional means for the acquisition of critical process variables, system monitoring and other tasks that are related to industrial process control [8]. These virtual sensors generally help regarding the unavailability of (real) hardware sensors in various industrial segments, thus allowing for less occurrence of failures and better control performance. Soft sensors are emerging as a viable alternative for the real-time monitoring of parameters that either lack a reliable measuring principle or are measured using expensive online sensors [12]. The purpose of a soft sensor is to estimate a variable that is not directly measured but that is related by a suitable model to several other process variables that are directly measured. Thus, for the context of applicability in WSS, the basis for the development of the soft sensor used is the ANNs (artificial neural networks). The option for the developed technique was based on some properties of the ANNs in which they were decisive for the adoption of this technique, such as: (a) no need for mathematical models; (b) generalizability; (c) pattern classification; and (d) learning, among others [7].

### 3.2. Indirect Measurements

Several technologies can be used to measure the main physical quantities in WSS. The flow rate, which is of greater interest in this work, can be measured using technologies such as electromagnetic and ultrasonic signals, among others. Regarding technologies that employ electromagnetic principles, associated equipment has higher acquisition and installation costs, depending on the installation diameter and, consequently, on the volume to be measured [5,6].

Other techniques can also be used to measure the flow, such as the equations that characterize the operation of a pump (pressure–flow curve). However, the use of this estimation method requires constant system calibrations, since they are based on the constructive characteristics of the pump, which wears out during operation, varying its characteristics over time [7]. Given the various studies presented, it is important to use techniques that eliminate (or mitigate) the situations, and indirect measurement techniques can be very useful in this regard.

In general, indirect measurements estimate the main variable (or quantity or magnitude) of an electrical signal obtained by directly measuring a secondary variable that is related to the main variable. The realization of an indirect measurement assumes a mathematical model that describes the association between the quantities involved and generally, the relationship between the secondary variable and the main variable is described by differential equations.

**Figure 1:**
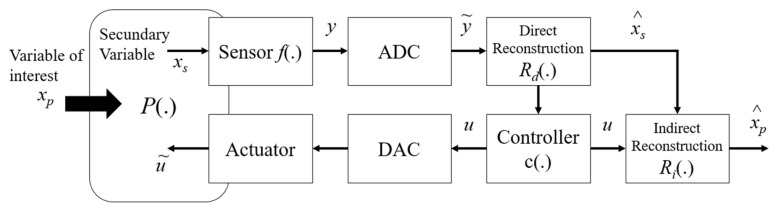
Generic diagram of a measurement feedback system adapted from [16].

Figure 1 illustrates a detailed flowchart of a feedback measurement system adapted from the model proposed by [16], in which the measurement means $P\left(.\right)$ is dynamically related to the primary variable, $x_{p}$, the secondary variable, $x_{s}$ and the actuation signal, u.

Using sensors to acquire input variables, the secondary variable $x_{s}$ is acquired and converted into an electrical signal y using the sensor function $f\left(.\right)$. Then, this signal is converted to a digital signal through an AD inverter providing a signal $\tilde{y}$, which is used to estimate the secondary quantity $x_{s}$, using the reconstruction function $R_{d}$. This last relationship corresponds to the inverse function of the sensor implemented in a discrete system. The signal ${\hat{x}}_{p}\;$ corresponds to the reconstruction of the signal coming from the input but indirectly from the controller $c\left(.\right)$ and the direct reconstruction $R_{d}\left(.\right)$. Equation (1) describes the conversion factor to obtain the $x_{s}\;$ signal [7].

(1) $$x_{s}=R_{d}\left(y\right)=f^{-1}\left(y\right)\;$$

The DA inverter and the actuators present are responsible for controlling the secondary variable $x_{s}\;$ at a certain reference value (setpoint). For this, the controller uses the measured values of the secondary variable together with the setpoint value, generating a discrete signal (u). This signal is converted into an analog signal (through a D/A inverter) and then through an actuator; this converted signal is applied to the $P\left(.\right)$ plant. Thus, after these procedures, the main variable is estimated in the indirect reconstruction block $R_{i}\left(.\right)$, using the values of the control signal. Equation (2) represents this estimation.

(2) $$x_{p}=R_{i}\left(x_{s},u\right)\;$$

The actuation signal modifies the dynamics of the plant $P\left(.\right)$ so that the response of the secondary signal $x_{s}\;$ follows the setpoint value; therefore, it will modify the dynamics in the estimation of the main variable $x_{p}$. The convergence speed of this estimate will depend on how the controller is designed.

### 3.3. Adaptive Control

In today’s language, the term adapt means to modify behavior according to new circumstances or situations. In the engineering context, adaptive control can be defined as a control technique that could change its behavior according to changes in parameters, in the dynamics of a process or by disturbances that affect this system [15]. Thus, a fixed gain controller (PID for example) is not an adaptive system. An adaptive controller must have the ability to update its control law by changing its gains (or parameters) in real time. In applications involving WSS, external parameters such as temperature, equipment wear and disturbances can alter the system’s operational dynamics. Thus, the use of controllers with static gains, in these scenarios, ends up not being convenient because they are tuned considering that the system is invariant in time and/or only for a specific operating range. Furthermore, the existence of noise and interference from different sources can directly influence the controller’s performance and, in the worst cases, lead to instability in the system [4]. To control these categories of system, controllers with adaptive gains stand out from those with static gains, as they could modify their parameters according to the changes suffered by the system [7,14].

#### Direct Adaptive Control

**Figure 2:**
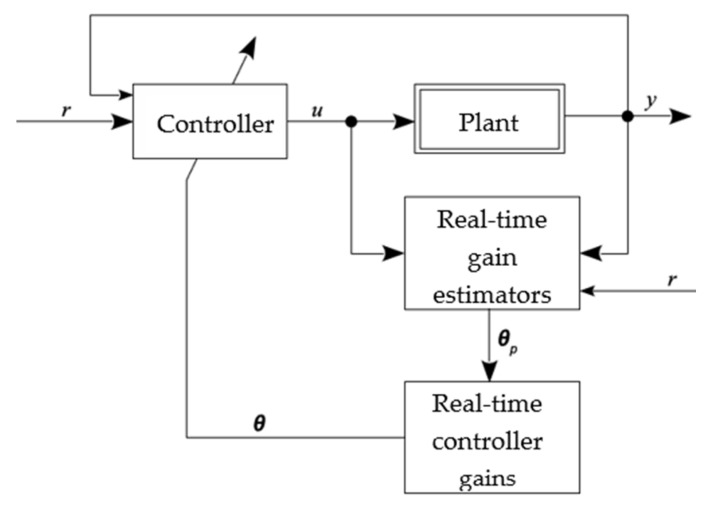
Direct adaptive controller.

Adaptive controllers can be divided into two large groups: direct and indirect. In the direct method, the controller gains are estimated directly from a pre-established reference model; that is, it is not necessary to carry out the identification of plant parameters [4]. Considering a reference signal *r* and an output *y*, in the direct method, the controller gains (vector *θ*) are estimated directly, usually from a pre-established reference model; that is, there is no need for direct estimation of the plant parameters. In the indirect method, the plant model is determined as a function of the unknown plant parameter vector, requiring a real-time estimator, using the input and output of plant signals. Therefore, the generated model is treated as true, and its parameters are used for the calculation of controller variables. Figure 2 illustrates the representation of a direct adaptive controller, which will be used for pressure control in this work.

For the direct adaptive control method, most works use the Adaptive reference model controller (MRAC). In this controller, the plant output signal is compared with a model output reference signal, generating a tracking error (ε). Using a cost function, the controller parameters are adjusted based on this error, causing the plant output signal to converge with that of the reference model. The matching condition is reached when ε is equal to zero. The controller parameters are adjusted based on this error, converging the plant output signal to that of the reference model. Generally, for this cost function, the MSE (mean squared error) is the standard adopted [4]. The MSE was adopted to perform the statistical analysis of error tracking in this work.

**Figure 3:**
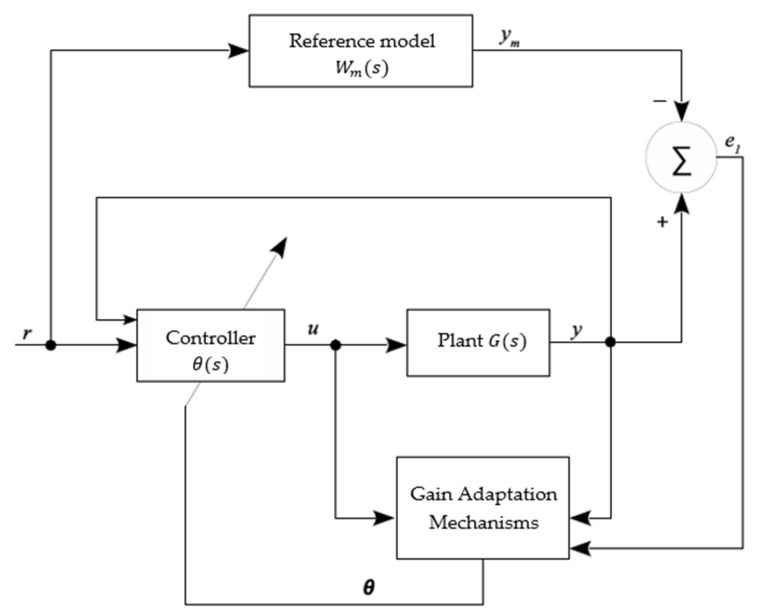
Adaptive controller by reference model.

Thus, a reference model control system is one in which the dynamic behavior of the closed-loop system is ideally identical to that of the reference model chosen $W_{m}\left(s\right)$ by the designer, as illustrated in Figure 3, so that the y sign follows the $y_{m}$ sign. That is, the performance specifications of the controller are defined by a predefined transfer function (in this case, $W_{m}$), considering a certain input signal r. From the input variables u and output y of the plant $G\left(s\right)$, a parameter vector θ is calculated in such a way that the tracking error $e_{1}$ is minimized. Thus, it is desired that the response *y* of the plant follows the response $y_{m}$ of the reference model $W_{m}\left(s\right)$ [15].

The IMRAC-PID controller (proportional–integral–derivative initialized model reference adaptive control) was developed by [4] and used in this research to promote pressure management in the system. This controlled pressure will be further detailed in the experiments of Section 4.6.3 and Section 4.6.4. The control action was useful to help the project objective through a subset of tests belonging to a larger set of validations (tests with and without controller action in the plant).

## 4. Proposed Methodology

### 4.1. Setup of Measurement

**Figure 4:**
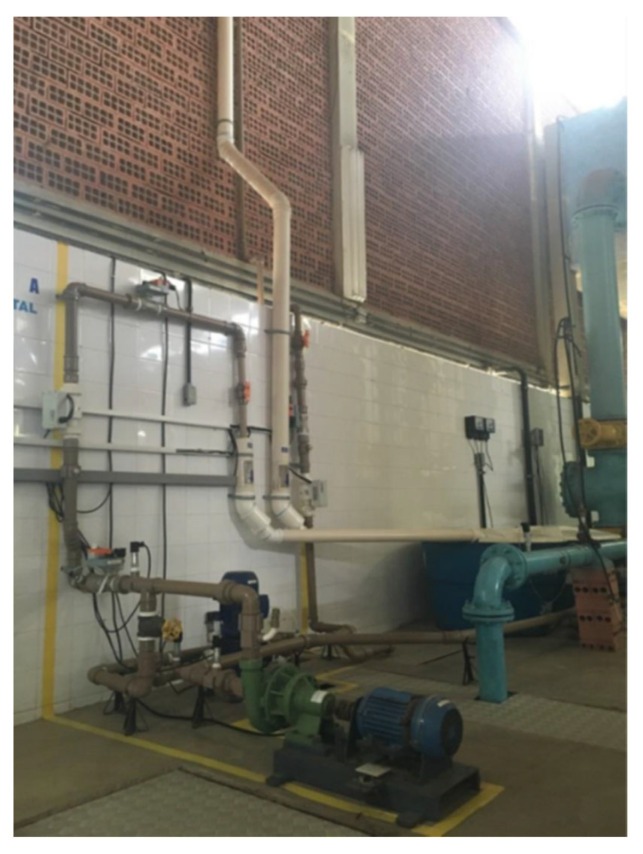
Experimental bench.

**Figure 5:**
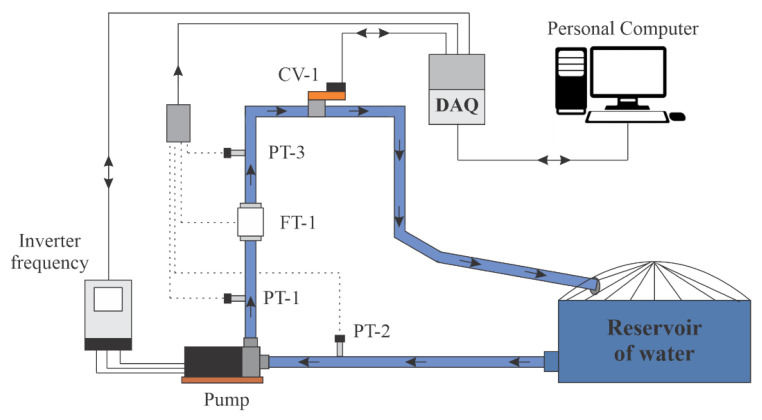
Bench schematic [4].

The experiments were carried out on an experimental bench set up on the premises of the Laboratory of Energy and Hydraulic Efficiency in Sanitation at the Federal University of Paraíba—LENHS/UFPB. The methodology described in this work can be applied to large WSSs, such as those existing in city water supply networks. The plant used in this research emulates a real WSS. However, for the purpose of validating the indirect flow estimation technique, the proportions corresponding to the physical elements adopted here correspond to those of an experimental bench. In this work, an experimental system was used, as illustrated in Figure 4, which emulates a water supply system with variable demand and Figure 5 illustrates the bench layout, respectively.

The pumping system was composed of a motor-pump set (three-phase, 220/380 V, 3 hp) which is made up of a centrifugal pump coupled to an electric motor. This structure was responsible for pushing water from the reservoir to the distribution system, which was composed of sensors and pipes (PVC). The pump’s rotation speed was controlled by a frequency inverter. In addition, at the outlet of the system was an automated proportional valve (PRV or CV-1), which served to emulate the variable water demand by regulating the cross-sectional area through which water flowed through the pipe. The electrical signals from the sensors in the form of a current (4–20 mA) were converted to voltage (0–10 V) by an electrical conditioning board. Afterward, the voltage levels were converted into a digital signal by a data acquisition system (DAQ) model NI-USB 6229, with a sampling frequency set at 10 samples/s. Finally, the digital signal was transmitting via USB to a personal computer (PC) for storage and digital processing. Next, application of the control algorithm was carried out; then, an actuation signal was generated and sent from the PC to the frequency inverter via DAQ. To validate the estimation technique presented in this work, the aging of the devices used, such as the natural wear of the pump, pipes and other elements belonging to the system, were not considered.

**Figure 6:**
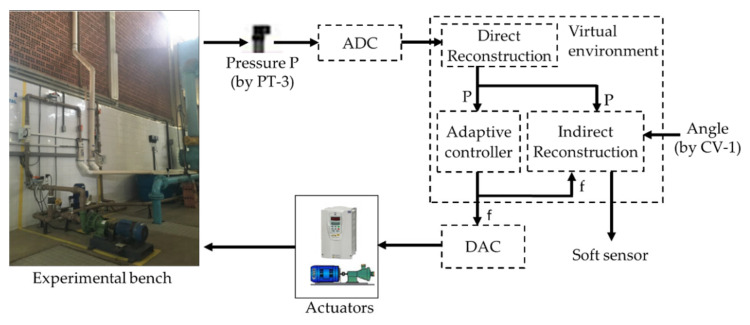
Feedback system for indirect flow measurement.

Figure 6 illustrates a schematic representation of the process topology used in the survey. Furthermore, it is possible to observe which specific signals were used by the estimator to measure the flow in the system. The choices of input variables for the controller—pressure and rotation frequency—were given by correlations they have: the pressure has a direct correlation with the frequency of the inverter (in this case the rotation frequency) and the angle of the CV-1. This last input constitutes an additional input (in addition to the pressure and rotation frequency) of the soft sensor.

The pressure signals (*P*) collected in PT-3 were converted into a digital signal by means of an analog-to-digital converter (ADC), the result of which is a signal that corresponds to the input port of the adaptive controller as well as the control block indirect reconstruction (soft sensor). The controller output was a frequency signal (*f*) acting for the variation in the CMB rotation as well as to compose one of the soft sensor inputs together with the CV-1 angle and the pressure (*P*). The frequency signal obtained at the controller output was converted into an analog signal, by means of a digital-analog converter (DAC), that served as an input for the frequency inverter actuation and, consequently, for the variation in the pump rotation speed.

### 4.2. Controller Structure

The structure of the adaptive controller developed by [4] and used in this work consists of the following elements: reference model in which the dynamic behavior of the closed-loop system is ideally identical to that of the transfer function chosen $\left(W_{m}\left(s\right)\right)$; plant that is the physical system to be controlled $\left(P\left(\theta^{*}\right)\right);$ controller is the proportional–integral–derivative (including $K_{p},\;K_{i}$ and $K_{p}$) with variable earnings (C); estimated plant that corresponds to the system identification function ($P_{e}$); parametric estimation mechanism implements estimation of plant parameters (*θ*) and adaptation mechanism of the controller that update controller’s gains ($\theta_{e}$).

**Figure 7:**
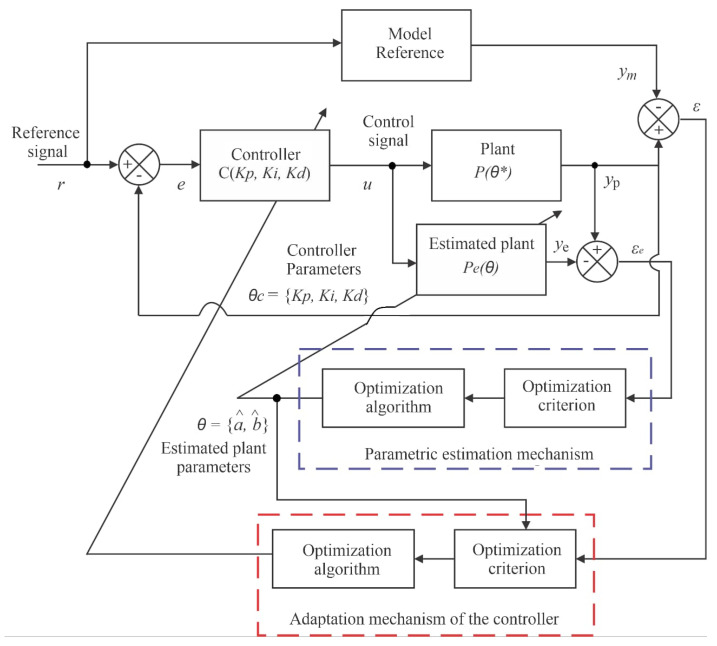
IMRAC-PID block diagram [4].

Figure 7 illustrates the block diagram of the control system [4].

The optimization criterion used in this work to evaluate the estimation mechanisms and parametric adaptation of the adaptive controller used was the MSE. The unknown parameter vector function $\theta^{*}$ was responsible for generating the plant model $P\left(\theta^{*}\right)$. A real-time estimator generated an estimate of *θ*(*t*) of *θ* at each instant of time *t*, calculating the input u and the output$\;y_{p}$. As a result of this processing, the estimation of the parameters *θ(t)* determined an estimated model, characterized by $P^{*}\left(\theta \left(t\right)\right)$*,* which corresponds to the true of the plant at time t. This result was used to determine the controller parameters or gain vector $\theta_{c}\left(t\right)$. The sentence that describes this representation can be visualized through Equation (3) [4].

(3) $$\theta_{c}\left(t\right)=F(\theta_{c}\left(t\right))$$

### 4.3. Applied Methodology

Equations commonly used for fluid analysis in water systems could be used to define the dynamic behavior of the system, together with the Darcy–Weisbach Equation. However, the equation’s form is a nonlinear and multivariable modeling of a complex solution and with approximations that limit its generalized application [17]. To overcome these disadvantages, this work proposes the use of artificial neural networks (ANNs) to compose the indirect estimation of the flow. The main advantage for using an ANN is the lack of mathematical models, mainly because it is a complex and multivariable problem, due to its generalization capacity, fault tolerance, self-learning, noise immunity and adaptability [13,17].

**Figure 8:**
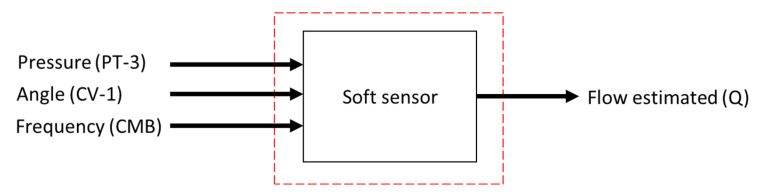
Diagram of Multi-layer Feedforward Perceptron Backpropagation ANN without feedback, with three input vectors and an output.

**Figure 9:**
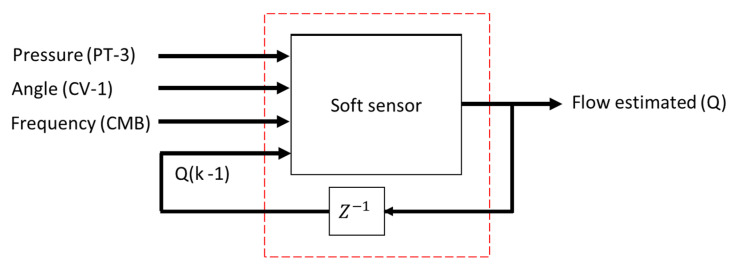
Diagram of ANN-NARX with feedback.

In this context, for performance comparison, this work addresses two ANN topologies to compose the indirect flow estimation block $R_{i}(.)$, as shown in Figure 8 and Figure 9. The first ANN contains three input vectors (frequency (f), angle of the valve CV-1 (a) and pressure measurement (P) and an output (flow (*Q*)), called multi-layer feedforward backpropagation ANN. The second ANN uses the same input and output vectors; however, the input vector is added by the feedback of the past value of the estimated flow $\left(Q\left(k-1\right)\right)$, called ANN nonlinear autoregressive exogenous (NARX). The choices of input variables for the controller—pressure and motor rotation frequency—were given by correlations they have; pressure has a direct correlation with the frequency (in this case the motor rotation frequency) and the CV-1 valve angle. This last input constitutes an additional input (in addition to the pressure and motor rotation frequency) of the soft sensor.

Table 1 contains the results of several tests performed to obtain the most optimized parameters for the ANNs proposed in this work. The fourth line (highlighted in bold) corresponds to the optimized parameters used in the ANNs.

The proposed ANNs have a hidden layer with eight neurons followed by an output layer with one neuron, determined empirically to find the network with the least error during the training period. The activation function adopted for the hidden layer is hyperbolic tangent sigmoid (tansig), and that of the output layer is linear. The training algorithm used was Levenberg–Marquardt (LM) for both proposed structures. The time spent to carry out the training of the two networks was also observed.

The parameters contained in Table 1 and which were used to optimize the elements of the ANNs are the MSE, which is normally used as a network performance indicator; gradient, that corresponds to an algorithm that seeks to minimize the MSE in order to obtain an optimized set of weights, which will end the training process, making the network able to produce acceptable output patterns (lowest value obtained was $1.32\times 10^{-9}$); and epoch, which corresponds to a complete cycle in which the neural network “visualized” all its data, among the parameters already mentioned.

### 4.4. Implementation of the Soft Sensor Using Artificial Neural Networks

**Figure 10:**
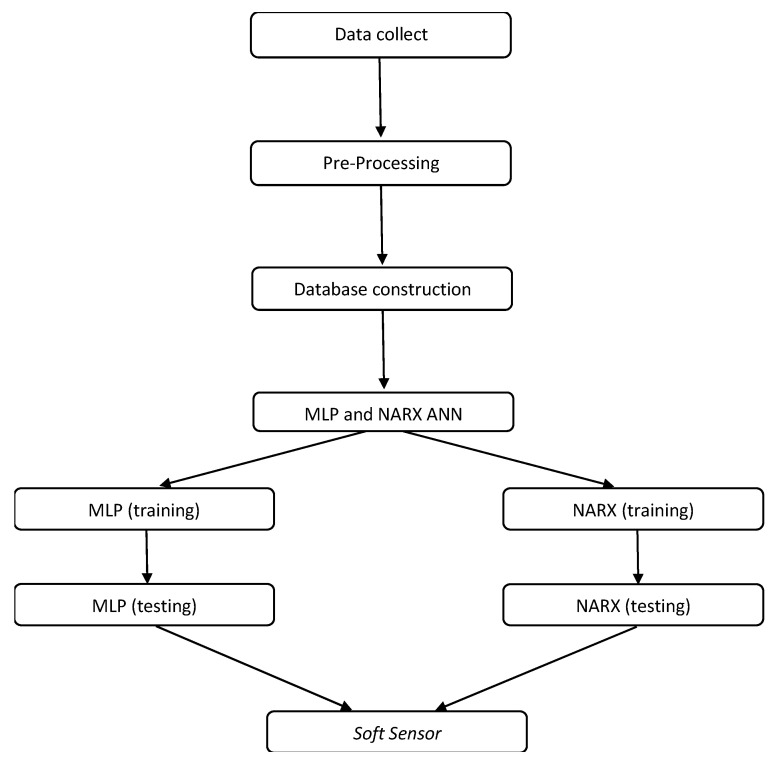
Flowchart for obtaining of the soft sensor.

For the implementation of the soft sensor, the Matlab software (v. 2020b) was used, its subsequent integration to the Supervisory System was implemented in Labview (v. 2017). The sequence of steps used to design the soft sensor can be visualized through a flowchart represented by Figure 10.

Data collection (step 1): this step corresponds to the acquisition of data from the instruments and equipment of the plant that will serve as inputs to the flow estimator. The data of interest are the rotation frequency of the motor; the opening (or closing) angle of the CV-1 valve; and the pressure relative of the plant (collected through sensor PT-3) and the flow (collected through sensor FT-1).

Pre-processing (step 2): the pre-treatment of data aims to detect and remove present anomalies to increase and improve their quality [18]. At this stage, the LOWESS (locally weighted regression scatter plot smoothing) data-smoothing technique was used for pre-treatment of these collected data.

Database construction (step 3): the collected data (step 1) were arranged in column-type vectors, separated according to each variable of interest to the estimator. This information will compose the ANN input parameter database. For each variable collected, about 70% of the first data obtained were separated for the ANN training step (described in step 4) and another number (about 30%) were used for the ANN testing step (also described in step 4). The criterion for dividing the data to be used in the training and testing phases of the networks was empirical.

**Figure 11:**
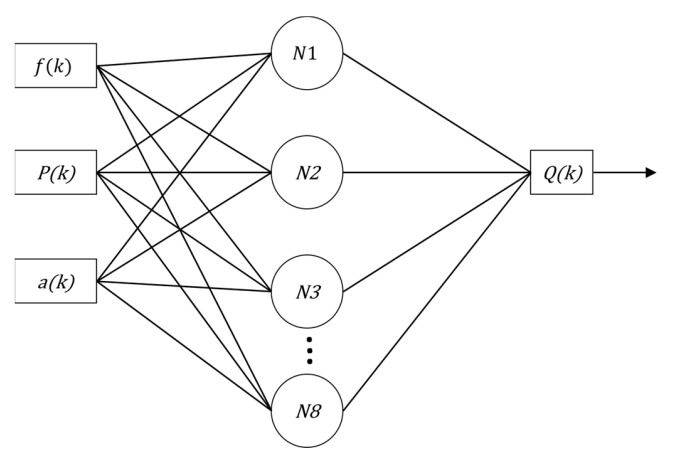
Diagram of Multi-layer Perceptron Feedforward Backpropagation ANN without feedback, with three input vectors, eight neurons and an output.

**Figure 12:**
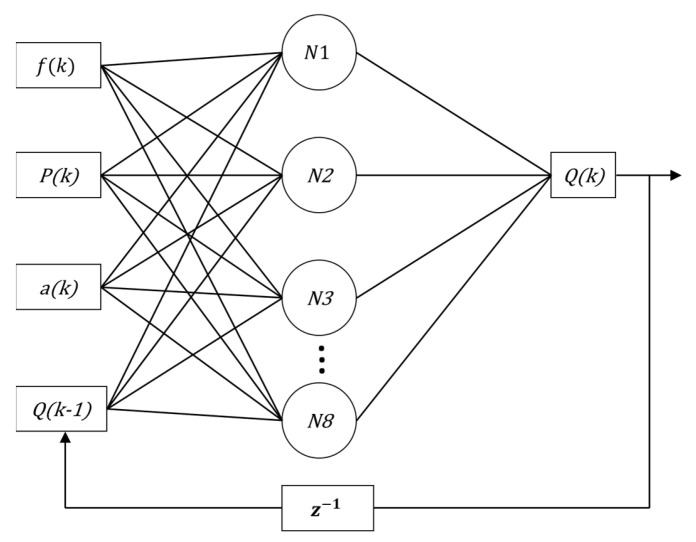
Diagram of NARX ANN with feedback, with four input vectors, eight neurons and an output.

MLP and NARX (step 4): This step corresponds, in fact, to the implementation of ANN networks: the multilayer feedforward perceptron backpropagation and the NARX (nonlinear autoregressive with exogenous inputs) and the result of these implementations is the soft sensor (step 5). Figure 11 and Figure 12 illustrate, respectively, the structures of the implemented MLP and NARX ANNs.

Two types of networks capable of performing the soft sensor function were implemented: one network using the multi-layer backpropagation ANN technique and the other using the nonlinear autoregressive ANN technique with exogenous inputs. From then on, tests were carried out with both networks to verify which one would be adopted to, in fact, act as a flow estimator on the experimental bench. Section 4.5 below, contains the experimental procedures for choosing, among the two ANNs, which one was to be the candidate to exercise the soft sensor function.

### 4.5. Tests for the Choosing of the Soft Sensor

The test was carried out with the objective of verifying which of the two developed soft sensors was the most efficient in terms of flow estimation when the system emulated the intermediate consumption demand in the experimental bench network. For this condition, the following premises were adopted for the collection of plant data: the pump started from rest (0 Hz) and then its rotation frequency varied in steps of 30 Hz, 40 Hz, 50 Hz, 60 Hz, 50 Hz, 40 Hz and 30 Hz every 3 min; the valves CV-1 and CV-3 remained with their opening angles always in the position of 45° each, while the valve CV-2 was in the position of 0° and the pressure (referring to the PT-3 sensor) was measured with the experimental system operating in an open loop. One of the estimator inputs was the CV-1 valve opening or closing angle. The CV-2 and CV-3 valves (angle CV-2 and angle CV-3), shown in the parameter Tables of some of the experiments performed, were auxiliary only (and not visible through Figure 5). They promote demand variation and simulate network disturbances, and they are not part of the soft sensor input parameters. Table 2 shows the parameters used for the tests related to test.

Regarding the data collection procedure, around 2160 samples were obtained. The sampling rate was fixed at 10 samples/s, in which the first 1500 samples obtained were used for training the ANNs and the remaining 660 were used for the test step. Input data (frequency, valve angle and system pressure) were pre-treated before being processed by the ANNs. Section 4.6 (and its subsections) contains the experimental procedures regarding NARX validation based on testing without the control action on the system and with the control action on the system.

### 4.6. Soft Sensor Validation

From the results presented in the previous section, only the soft sensor implemented through the ANN of the NARX type was adopted for the validation tests of the flow estimator. For this new category of tests, the monitoring of the actual flow (measured through the electromagnetic flow sensor FT-1) versus estimated flow was carried out simultaneously and during the real-time performance of the tests. For this, the supervisory implemented in LabVIEW was used for parameterization and control of the plant and the soft sensor, developed in Matlab, whose code was inserted into the supervisory. The tests described below validate, in fact, the implemented soft sensor, whether with or without the controller acting on the system.

#### 4.6.1. Soft Sensor Validation without the Action Controller: Testing A

The validation test was carried out with the objective of verifying the insertion of sudden consumption demands in the water supply network without the controller acting to verify the performance of the soft sensor in this situation. Sudden demands on water supply systems can be caused by obstructions or leaks in the network, among other possibilities. For this condition, the following premises were adopted: the pump started from rest (0 Hz), and from then on, the rotation frequency of the motor was randomly modified within an interval of 30 to 60 Hz, with a duration of 2 min each modification; the three control valves had their opening angle varied within a range of 0° to 75° with a duration of 2 min each modification and the pressure were measured with the experimental system operating in an open loop.

Table 3 shows the parameters used for the tests related to test.

#### 4.6.2. Soft Sensor Validation without the Action Controller: Testing B

This test aimed to verify the performance of the soft sensor when the three inputs (PT-3 pressure, CV-1 valve and the rotation frequency of the motor) were subjected to outliers. Table 4 shows the parameters used for the tests related to test. Another common challenge for developing a practice soft sensor is its noise reliability [18]. Type signals with different amplitudes and without the controller action. For this test, the following assumptions were adopted: the rotation frequency of the motor variation starting at rest (0 Hz) and being switched to 30 Hz, 40 Hz, 50 Hz, 60 Hz, 50 Hz, 40 Hz and 30 Hz. The time adopted for each frequency change was one minute; the pressure acted freely, being measured following the frequency variation in the motor, and the valve CV-1 was at 0° while CV-2 and CV-3 were at 45° during test execution.

#### 4.6.3. Soft Sensor Validation with the Action Controller: Testing C

This test aimed to verify the performance of the soft sensor by estimating the flow when the controller was subject to change in the desired pressure value. For this test, the following assumptions were considered: $K_{p}=K_{i}=K_{d}=\alpha_{1}=\alpha_{2}=\alpha_{3}=0.01$. The parameters $K_{p}$, $K_{i}$ and $K_{d}$ represent the proportional, integral and derivative gains of the IMRAC-PID controller, respectively. The constants $\alpha_{1},\;\alpha_{2}$ and $\alpha_{3}$ represent the estimation parameters of the controller’s model $W_{m}\left(s\right)$ design to the pressure control, from the same plant used in this work [4] and visualized through Figure 3 (gain adaptation mechanisms block); the CV-2 and CV-3 valves remained with their opening angles always at the 45° position, while the CV-1 valve was at the 0° position and the pump was initially at rest (0 Hz); the desired pressure values were equal to 8, 10, 12, 14, 16, 14, 12 and 10 mH_2_O with an approximate duration of 60 s each SP from the stabilization of the first one, and the rotation frequency of the motor followed the pressure setpoint.

#### 4.6.4. Soft Sensor Validation with the Action Controller: Testing D

This test aimed to verify the performance of the soft sensor when the three inputs (pressure in PT-3, valve CV-1 and the rotation frequency of the motor) were subjected to signal-type noise with different amplitudes. This scenario represents one of the worst situations that can happen to the estimator’s input elements. For this test, the following assumptions were adopted: $K_{p}=K_{i}=K_{d}=\alpha_{1}=\alpha_{2}=\alpha_{3}=0.01;$ the CV-2 and CV-3 valves remained with their opening angles always in the 45° position, while the CV-1 valve was in the 0° position and the pump was initially at rest (0 Hz); the pressure setpoint was fixed at 10 mH_2_O throughout the test; the rotation frequency of the motor followed the pressure setpoint from the beginning until its stabilization and the spurious (continuous noise type) signals inserted into PT-3, CV-1 and rotation frequency of the motor had a duration of approximately 100 s.

## 5. Experimental Results

In this section, the results of the proposed methodology are presented, evaluating the controller performance for the secondary variable (pressure), and the multi-layer feedforward backpropagation ANN and ANN-NAXR networks, presented in Section 4.4, making comparisons for robustness and dynamic response. The results were collected in the water supply system installed in the Laboratory of Hydraulic and Energy Efficiency in Sanitation at the Federal University of Paraíba (LENHS/UFPB), as shown in Figure 4.

### 5.1. Results about the Choosing of the Soft Sensor

**Figure 13:**
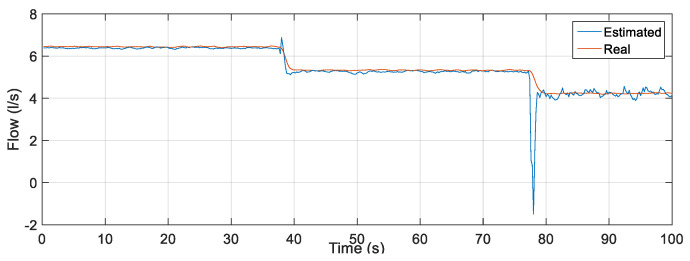
Measurement and estimation of flow during ANN testing using Multi-layer Feedforward Backpropagation.

Initially, the MLP backpropagation ANN was examined for the test phase of the network using the input data: rotation frequency of the motor, CV-1 angle and pressure measured in PT-3. The estimated output was the flow. Figure 11 illustrates the flow measured through an electromagnetic flow sensor (FT-1) and the estimated flow resulting from the test phase for the MLP backpropagation ANN. There is a measurement deviation regarding the flow rate of up to 5.5 L/s (outlier) between the estimated and measured values along the curves visualized in Figure 13. This discrepancy occurs due to the ANN’s lack of knowledge of the system dynamics, that is, the lack of knowledge of the flow behavior in the previous samples.

**Figure 14:**
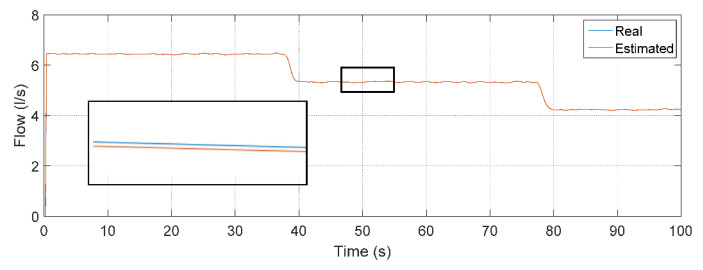
Measurement and estimation of flow during ANN testing using NARX.

To quantify the tracking of the error obtained through the results obtained from the data that composed the curves in Figure 13, some statistical measures referring to the estimator based on MLP were determined: mean of the relative error, whose result was 0.0248%; maximum relative error equal to 20.9210%; and standard deviation of the relative error equal to 1.0281%. To compare the performance between the estimators, the simulation for the NARX test phase was also performed, using the same MLP input data. However, the flow data resulting from the processing of PT-3, frequency and CV-1 inputs was reinserted (feedback) as an additional Q(k − 1) delayed input. This feedback introduces the dynamics to the system so that network learning is constantly optimized. Figure 14 illustrates the output regarding the estimated flow using the estimator developed in NARX.

To quantify the tracking of the error through the data that made up the graphs illustrated in Figure 14, statistical measures were determined for the NARX: mean relative error, whose result was 0.0015%; maximum relative error equal to 0.3090%; and standard deviation of the relative error equal to 0.0247%. Table 5 shows a comparison between the calculated measurements for the two implemented soft sensors, showing the efficiency with respect to the accuracy and reliability of the estimation of the flow using a NARX in the simulation phase.

### 5.2. Soft Sensor Validation without the Action Controller: Testing A

**Figure 15:**
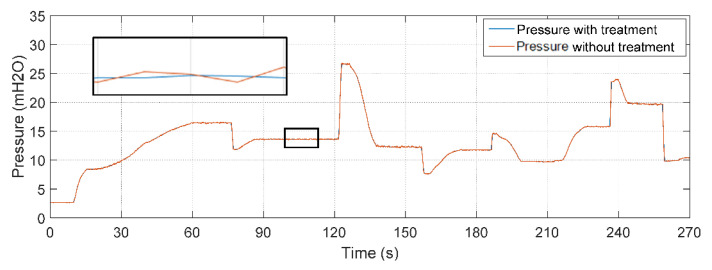
Pressure in PT-3 with and without data pre-processing.

Figure 15 illustrates the curve corresponding to pressure in PT-3 (with and without raw data processing) and its correspondence for each frequency change entered in this validation test. Frequency variation was randomly defined within a range of 30 Hz to 60 Hz.

**Figure 16:**
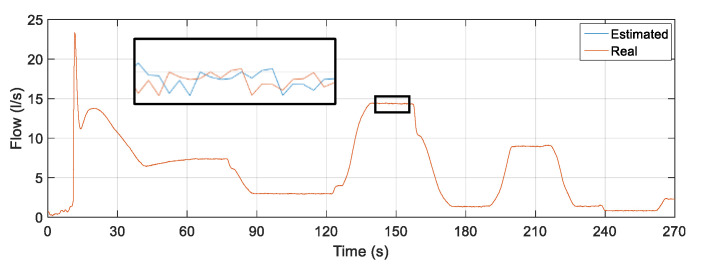
Estimated and real Flow: Testing A.

The result of the comparison between the actual value of the flow measured through the FT-1 electromagnetic instrument and the flow estimator for the conditions mentioned in this test is illustrated in Figure 16. It is observed that the estimated value curve is very close to the flow curve with relative error average equal to 0.0054% (values measured by the electromagnetic flux transducer), as highlighted. The oscillations present at the beginning are due to the previous presence of water in the pipes.

It is observed that, for this test, it is not enough for the motor to have increasing frequency values for the pressure to also rise proportionally. Now, in addition to this factor, there is the combination of opening and/or closing of the three control valves that can influence both the estimated and actual flow curves, sometimes acting in an inversely proportional way to the increase in pressure in the system. To quantify the error tracking through the data that composed the graphs illustrated in Figure 16, some statistical measures related to the relative error were determined by comparing the actual versus estimated flow curves. The results obtained are shown in Table 6.

When observing the data referring to Table 6, it is observed that the mean and standard deviation of the tracking relative error have values much lower than 1%, which infers good performance by the soft sensor. It is important to note that all data were considered for the statistical analysis of error tracking; therefore, the maximum error obtained this relatively high value. Still in relation to this statistical measure, its result from the initial oscillations presents in the system resulting from the previous presence of water in the pipes. However, they do not interfere with the measurement performance of the soft sensor that adequately measured the flow for any given conditions.

### 5.3. Soft Sensor Validation without the Action Controller: Testing B

**Figure 17:**
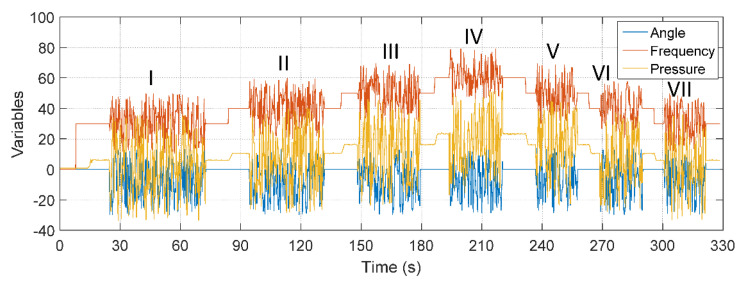
Inputs with noise in PT-3, CV-1 and pump.

Figure 17 illustrates the signal obtained for the three inputs with distinct random noises with Gaussian distribution in each one of them. The noises entered for this specific test are made up of signals of a Gaussian nature. They were divided into segments, each denoted by a Roman numeral, and each has a reference amplitude (constant value) added to a random value within a specific range. The nature of each noise segment used in this test can be seen in Table 7.

**Figure 18:**
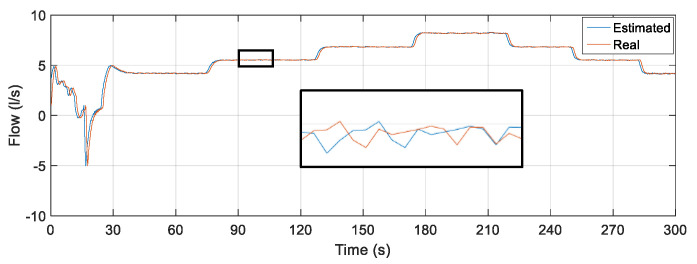
Estimated and real flow: Testing B.

The result of the comparison between the actual value of the flow measured through the FT-1 electromagnetic instrument and the flow estimator for the conditions mentioned in this test is shown in Figure 18.

The curve of the value estimated by the soft sensor is very close to the flow curve (values measured by the electromagnetic flux transducer), as highlighted. Quantitatively and in standardizing tests for all validations, some statistical measures were taken regarding error tracking, and the results obtained are shown in Table 8.

The efficiency of the soft sensor allows the indirect measurement of flow to be carried out efficiently even with the possibility of spurious signals at its three inputs. It is observed, from the comparison between the error-tracking statistical measures, that all results have values much lower than 1%, validating the performance of the soft sensor. Regarding the maximum error obtained with the analysis of all points on the curves, its result of 2.81% results from the initial oscillations already described and from the consideration of all the points that form the analyzed curves.

### 5.4. Soft Sensor Validation with the Action Controller: Testing C

**Figure 19:**
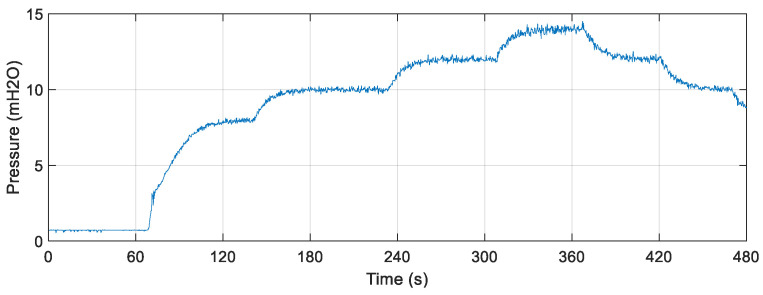
Controlled plant responses with abrupt variations in consumption demand.

Figure 19 illustrates the controlled pressure curve with fixed setpoint increments and decrements. It is observed that the plant took about 135 s for its signal to reach the first value of the adopted reference model (8 mH_2_O). The larger the above-mentioned parameters, the longer the time needed to reach the setpoint value. On the other hand, the performance of the system was better in terms of a smoother response.

**Figure 20:**
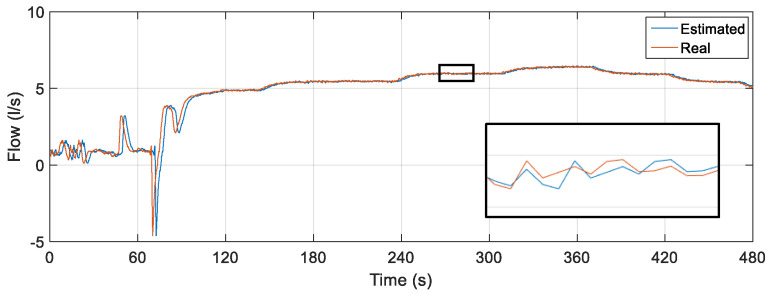
Estimated versus actual flow with the plant controlled for different desired pressure values.

The result of the comparison between the real value of the flow measured through the electromagnetic instrument FT-1 and the estimator through the soft sensor is illustrated in Figure 20. The soft sensor was able to properly estimate the flow. The oscillations in the first moments of time are the effects of the presence of water in the pipes before the controller is activated. When the signal is stabilized, the elimination of these transients is observed. However, even with initial sudden oscillations, it is already possible to verify the performance of the soft sensor following the actual flow value in any situation.

For quantitative analysis, some statistical measures related to error tracking were determined by comparing the data that composed the curves in Figure 18. The results obtained are shown in Table 9.

When comparing the mean and standard deviation of the tracking error, it is observed that both have values less than 1% (especially the mean), which infers good performance of the soft sensor. In relation to the maximum error (4.21%), its result is caused by the initial oscillations present in the system as well as from the statistical analysis that considered all points on the curve (including the initial oscillations of the system) and not only during the steady state. However, they do not interfere with the measurement performance of the soft sensor.

### 5.5. Soft Sensor Validation with the Action Controller: Testing D

The noises entered for this specific test are made up of signals of a Gaussian nature, and each has a reference amplitude (constant value) added to a random value within a specific range. The nature of each noise segment used in this test can be seen in Table 10.

**Figure 21:**
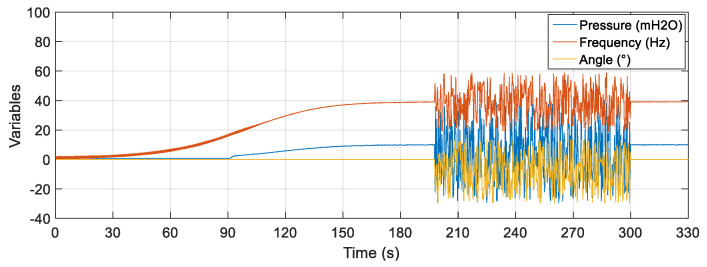
Rotation frequency, CV-1 valve angle and PT-3 pressure input signals with noise.

Figure 21 illustrates the noisy curves at the rotation frequency of the motor, valve CV-1 and pressure sensor PT-3. Random and distinct noises were inserted for each input of the estimator with a duration of 200 s (starting at 100 s and ending at 300 s). The noises remained until the approximate time of 300 s.

**Figure 22:**
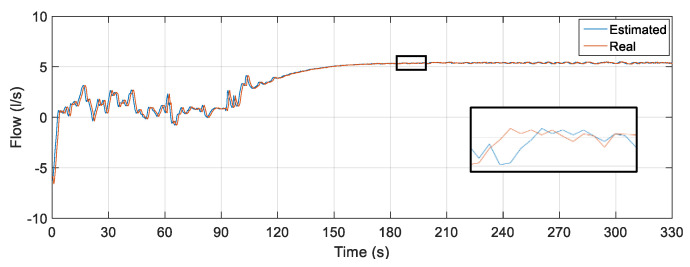
Estimated versus actual flow rate for inserting noise into the three soft sensor inputs.

The result of the comparison between the actual value of the flow measured through the FT-1 electromagnetic instrument and the flow estimator is illustrated in Figure 22.

The soft sensor was able to adequately estimate the flow in the worst-case scenario: the insertion of spurious signals simultaneously in all its inputs. As in the previous tests, the oscillations present in the first moments of time are a consequence of the presence of water in the pipes before the controller is activated. However, even with initial oscillations and extreme input situations, a satisfactory performance of the soft sensor is observed, following the actual flow rate in such situations. To analyze the data referring to the points that form the curves illustrated in Figure 22, some statistical measures related to the error tracking were determined for the curve (complete curve) as well as for the section referring only to the time instant of insertion of noises. The results obtained are shown in Table 11.

When comparing the error-tracking statistical measures, it is observed that all results have values well below 1%, which induces satisfactory performance by the soft sensor even with the insertion of noise in its three inputs. Concomitant spurious signals in the three inputs of the flow estimator correspond to one of the worst scenarios to verify the robustness of the virtual instrument. Regarding the maximum error obtained with the analysis of all points of the curves, its result, although with a low value, is caused by, as in the previous tests, the initial oscillations already described and the consideration of all the points that form the analyzed curves and not just the stabilized part of the curves upon reaching the steady state.

## 6. Discussion of Results

The validation tests were carried out with the developed soft sensor (NARX), chosen from the test results. These validation tests were categorized into tests without the presence of the controller acting on the system and with the presence of the controller acting on the system. The first test performed, without the controller’s action, whose test was categorized as “Validation A”, aimed to verify the insertion of sudden consumption demands in the water supply network without the controller’s action to verify the performance of the soft sensor in this situation. It was verified that the soft sensor was able to estimate the flow as expected when compared to the real FT-1 instrument. It was verified that the mean and standard deviation of the tracking error have values much lower than 1%, which infers good performance by the soft sensor. The other validation test was performed by inserting spurious signals into all inputs of the flow estimator. As a result, it was observed that the mean of the tracking relative error as well as the standard deviation of the tracking error resulted in values much lower than 1%. Only the maximum error value had a value above 1%. However, this was due to the previous presence of water in the pipes. Even in the face of adversities, the efficiency of the soft sensor allowed the indirect measurement of flow to be carried out satisfactorily in the tests without the presence of the controller acting in the plant.

The other soft sensor validation tests were performed by the controller in the plant. The controller used was the indirect adaptive type by reference model with PID (IMRAC-PID). The first validation test aimed to verify the performance of the soft sensor by estimating the flow when the controller was subject to change in the desired pressure value. Error-related statistical parameters (based on the tracking error using the MSE criterion) were obtained as a comparison between the estimated flow curve versus the actual flow through an electromagnetic meter. Another test was also carried out by inserting spurious signals in all its inputs to verify the performance of the virtual sensor in the face of these adversities. An average relative error and a standard deviation well below 1% were obtained and the highest value related to the maximum error obtained in only one of the tests performed occurred due to initial oscillations in the estimation curve in the first moments of flow measurement. This is because of the presence of water in the pipes. However, even with initial sudden oscillations, it was possible to verify the performance of the soft sensor following the actual flow value in any situation of all the tests performed.

## 7. Conclusions

The main objective of this work was the implementation of a virtual instrument, called a soft sensor, capable of measuring the flow in a water supply system. This virtual instrument replaces the use of a physical instrument with the advantages of significantly reducing acquisition costs as well as eliminating the operational complexity of a physical installation, depending on the measurement point of interest. For the design of this soft sensor, two structures were designed, using artificial neural network approaches belonging to the field of artificial intelligence: a multilayer ANN based on learning called multi-layer perceptron (ANN MLP) and an ANN based on output feedback, called neural network auto-regressive with exogenous inputs (NARX). The advantages of choosing and using ANNs are the dispensing of complex mathematical models used in other techniques that often limit the real scope of the system due to many considerations/restrictions to be carried out; the ability to learn and generalize applications regarding ANNs; and the adaptability capacity, among other advantages.

To evaluate the performance of the ANNs in the flow estimation process, the two designed networks (MLP and NARX) were implemented in the Matlab programming environment, where they went through the training and testing phases. After the test process, the NARX-type ANN was adopted to estimate the flow in a water supply system through an experimental platform, located in the Laboratory of Energy and Hydraulic Efficiency in Sanitation (LENHS/UFPB). The estimated flow values obtained by NARX were compared with values obtained through a real electromagnetic flow sensor present in the system. The validation tests using only the NARX network had as their objective the performance of the soft sensor being able to measure the flow in real time, when compared to a flow signal coming from an electromagnetic flow sensor without the action of the controller in the plant. It was observed that without the control action on the plant, the results obtained regarding the statistical parameters mean relative error and standard deviation of the relative error presented values much lower than 1% for the situation of insertion of disturbances in the system, as well as when there was insertion of outliers. This shows the efficiency of the flow estimation technique for the presented cases (without controller).

Pressure control in pumping systems is essential for reducing real losses and increasing energy efficiency, and it was verified that the adaptive controller behaved adequately. For tests where there was a control action acting on the system, the results obtained regarding the statistical parameters mean error, maximum error and standard deviation of the error (relative values) presented values much lower than 1% for the situation of insertion of disturbances in the system as well as when there was insertion of noise in the plant. This shows the efficiency of the flow estimation technique for the cases.

In the experimental bench used to validate the flow estimation through the soft sensor, only one reference reservoir was used. However, there is another reservoir coupled to the system, but not used in this research nor visualized through Figure 5. However, because they are two coupled reservoirs, their levels varied as the water was pumped through the system and the method estimation could also be applied to systems with different levels (different topographical heights). The absence of a level gauge in the reservoir used in this research made the validation unfeasible in case the pumping pressure was variable at the pump suction point.

In future research, other control techniques may be used, such as neural or neural-fuzzy control, which do not require prior knowledge of plant dynamics; the evaluation of other topologies of artificial neural networks such as ARMAX or convolutional neural networks, for example; and the use of other input parameters of a WSS and the use of autoencoders (or another technique) to reconstruct and correct measurements. Another suggestion for future work would be the application of the indirect flow estimation technique in real water supply systems. The large variations (consumption demand, presentation, etc.) that normally occur in real systems would serve as a very robust validation of the technique presented in this work.

## Figures and Tables

**Table 1:** Tests performed to obtain the optimized parameters of the ANNs.

MSE	Gradient	Epoch	Training Algorithm	Number of Hidden Layers	Number of Neurons	Activation Function
5.589 × 10^−12^	$6.12\times 10^{-6}$	1000	LM	1	5	tansig
7.313 × 10^−11^	$9.61\times 10^{-6}$	1000	LM	1	6	tansig
1.383 × 10^−11^	$3.68\times 10^{-6}$	1000	LM	1	7	tansig
**1.639 × 10^−10^**	$1.32\times 10^{-9}$	**1000**	**LM**	**1**	**8**	**tansig**
3.214 × 10^−10^	$3.30\times 10^{-6}$	1000	LM	1	9	tansig
3.434 × 10^−10^	$2.02\times 10^{-7}$	1000	LM	1	10	tansig
3.511 × 10^−9^	$3.47\times 10^{-5}$	1000	LM	1	11	tansig
1.313 × 10^−10^	$3.11\times 10^{-6}$	1000	LM	1	12	tansig
6.112 × 10^−10^	$2.68\times 10^{-6}$	1000	LM	1	25	tansig

**Table 2:** Parameters used for the test for choosing of the soft sensor.

Rotation Frequency (Hz)	Pressure (mH_2_O)	Angle CV-1 (°)	Angle CV-2 (°)	Angle CV-3 (°)
30	7.93	45	0	45
40	12.13	45	0	45
50	17.23	45	0	45
60	24.21	45	0	45
50	17.62	45	0	45
40	12.09	45	0	45
30	7.86	45	0	45

**Table 3:** Parameters used for the test of the soft sensor validation: testing A.

Rotation Frequency (Hz)	Pressure (mH_2_O)	Angle CV-1 (°)	Angle CV-2 (°)	Angle CV-3 (°)
50	4.9	41	35	72
41	1.4	61	18	77
60	6.3	9	6	52
39	3.6	73	68	30
45	4.3	20	15	52
46	14.5	21	76	48
58	18.5	3	84	14
35	7.2	50	51	64
30	5.4	11	35	31

**Table 4:** Parameters used for the test of the soft sensor validation: testing B.

Frequency (Hz)	Tags	Insertion Outliers (s)	Pressure (mH_2_O)	Angle CV-1 (°)	Angle CV-2 (°)	Angle CV-3 (°)
30	I	10 to 50	5.91	0	45	45
40	II	15 to 30	10.55	0	45	45
50	III	8 to 40	16.90	0	45	45
60	IV	10 to 55	23.17	0	45	45
50	V	20 to 40	16.35	0	45	45
40	VI	15 to 45	10.66	0	45	45
30	VII	5 to 55	6.34	0	45	45

**Table 5:** Ratio between statistical measures: choosing of the soft sensor.

Mean MLP BP/NARX ANN	Maximum Error MLP BP/NARX ANN	Standard Deviation MLP BP/NARX ANN
16.6173%	67.7072%	41.5573%

**Table 6:** Ratio between statistical measures: testing A.

Mean	Maximum Error	Standard Deviation
0.0054%	19.2742%	0.7384%

**Table 7:** Noises inserted into the soft sensor inputs: testing B.

Tags	Reference Range Angle (°)	Angle Limits (°)	Reference Range Pressure (mH_2_O)	Pressure Limits (mH_2_O)	Reference Range Frequency (Hz)	Frequency Limits (Hz)
I	0	−30 to 10	6	−35 to 35	30	10 to 50
II	0	−30 to 10	10	−30 to 40	40	22 to 60
III	0	−30 to 10	18	−25 to 50	50	30 to 70
IV	0	−30 to 10	22	−18 to 30	60	45 to 80
V	0	−30 to 10	18	−25 to 45	50	30 to 58
VI	0	−30 to 10	10	−30 to 40	40	22 to 58
VII	0	−30 to 10	6	−30 to 35	30	15 to 50

**Table 8:** Ratio between statistical measures: testing B.

Error Average	Maximum Error	Standard Deviation
0.0059%	2.8093%	0.3763%

**Table 9:** Statistical measures: testing C.

Error Average	Maximum Error	Standard Deviation
0.0081%	4.2124%	0.4138%

**Table 10:** Noises inserted into the soft sensor inputs: testing D.

Variable	Reference	Range Limits
Angle (°)	0	−30 to 10
Pressure (mH_2_O)	10	−30 to 40
Frequency (Hz)	39	20 to 58

**Table 11:** Statistical measures: testing D.

Corresponding Excerpt	Mean Error	Maximun Error	Standard Error Deviation
Just noises	2.3618 × 10^−^^4^%	0.1232%	0.0481%
Full curve	0.0306%	1.6982%	0.3510%

## Data Availability

Data sharing not applicable or no new data were created or analyzed in this study. Data sharing is not applicable to this article.

## Abbreviations

The following abbreviations are used in this manuscript:

ANN	Artificial Neural Network
DAQ	Data Acquisition System
FT	Flow Transducer
IFPE	Federal Institute of Pernambuco
IMRAC-PID	Indirect Adaptive Control
LENHS	Laboratory of Energy Efficiency and Hydraulics in Sanitation
MRAC	Reference Model Adaptive
MSE	Mean Squared Error
MLP BP	Multilayer Perceptron Backpropagation
NARX	Nonlinear Autoregressive with Exogenous Inputs
NI	National Instruments
PC	Personal computer
PID	Proportional–Integral–Derivative
PT	Pressure Transducers
PVC	Polyvinyl chloride
SP	Setpoint
UFPB	Federal University of Paraiba
USB	Universal Serial Bus
CV-1	Automated Proportional Valve (main valve)
CV-2	Automated Proportional Valve (auxiliar valve)
CV-3	Automated Proportional Valve (auxiliar valve)
